# Clinical heterogeneity of *PLA2G6*-related Parkinsonism: analysis of two Saudi families

**DOI:** 10.1186/s13104-016-2102-7

**Published:** 2016-06-07

**Authors:** Saeed A. Bohlega, Bashayer R. Al-Mubarak, Eman A. Alyemni, Mohamed Abouelhoda, Dorota Monies, Abeer E. Mustafa, Dania S. Khalil, Sara Al Haibi, Hussam Abou Al-Shaar, Tariq Faquih, Mohamed El-Kalioby, Asma I. Tahir, Nada A. Al Tassan

**Affiliations:** Department of Neurosciences, King Faisal Specialist Hospital and Research Center, P.O Box 3354, Riyadh, 11211 Saudi Arabia; Behavioral Genetics Unit, Department of Genetics, King Faisal Specialist Hospital and Research Center, P.O Box 3354, Riyadh, 11211 Saudi Arabia; Department of Genetics, King Faisal Specialist Hospital and Research Center, P.O Box 3354, Riyadh, 11211 Saudi Arabia; Saudi Human Genome Project, King Abdulaziz City for Science and Technology, Riyadh, Saudi Arabia

**Keywords:** *PLA2G6*, Parkinsonism, Saudi patients

## Abstract

**Background:**

Recessive mutations in *PLA2G6* have been associated with different neurodegenerative disorders, including infantile neuroaxonal dystrophy, neurodegeneration with brain iron accumulation and more recently, early-onset dystonia parkinsonism.

**Method:**

Targeted-next generation sequencing using a custom Neurology panel, containing 758 OMIM-listed genes implicated in neurological disorders, was carried out in two index cases from two different Saudi families displaying early-onset levodopa-responsive Parkinsonism with pyramidal signs and additional clinical features. The detected mutations were verified in the index cases and available family members by direct sequencing.

**Results and conclusion:**

We identified a previously described *PLA2G6* homozygous p.R741Q mutation in three affected and two asymptomatic individuals from two Saudi families. Our finding reinforces the notion of the broadness of the clinical spectrum of *PLA2G6*-related neurodegeneration.

**Electronic supplementary material:**

The online version of this article (doi:10.1186/s13104-016-2102-7) contains supplementary material, which is available to authorized users.

## Background

Oxidative stress is considered a key pathophysiological mechanism underlying dopaminergic neuronal loss in Parkinson’s disease (PD). Excess iron is one main contributor to oxidative stress that promotes the formation of harmful free radicals via the Fenton reaction. High iron content, in addition to other factors, including the presence of reactive oxygen species (ROS)-generating enzymes and dopamine oxidation, renders dopaminergic neurons of the substantia nigra vulnerable to oxidative stress [1]. Another source of ROS in the brain is the metabolism of arachidonic acid, which is released from membrane phospholipids by the hydrolytic activity of calcium-independent group VI phospholipase A_2_ (iPLA_2_-VI) [2].

Recessive mutations in *PLA2G6* [MIM 603604], the gene encoding (iPLA_2_β/iPLA_2_-VI), have been associated with different neurodegenerative disorders, including infantile neuroaxonal dystrophy (INAD), neurodegeneration with brain iron accumulation (NBIA) and more recently, early-onset dystonia parkinsonism [3]. The involvement of iPLA_2_-VI in oxidative stress [2], the identification of *PLA2G6* mutations in patients with parkinsonian features [3], and the presence of α-synuclein Lewy body pathology in five dystonia-parkinsonism cases with *PLA2G6* genetic abnormalities [4] suggest a possible role for this gene in PD pathogenesis.

Utilizing targeted-next generation sequencing (NGS) of 758 OMIM-listed neurological disorders-associated genes, we identified a homozygous p.R741Q mutation previously described in *PLA2G6* in three cases with early-onset Parkinsonism [MIM 612953] displaying additional clinical features. Our finding reinforces the notion of the broadness of the clinical spectrum of *PLA2G6*-related neurodegeneration.

## Methods

### Subjects

A total of 17 individuals (3 affected and 14 unaffected) from two families originating from the Eastern province of Saudi Arabia were recruited after obtaining written informed consent from all study subjects for participation in the study and for the publication of their genetic and clinical data. This study was approved by the King Faisal Specialist Hospital and Research Center (KFSHRC) IRB, Research Ethics Approval Committee (RAC# 2110035).

### Clinical assessment

Standardized clinical investigation was undertaken in all patients by senior neurologists. Neuroimaging examinations were performed using Axial brain fluid attenuated inversion recovery (FLAIR) MRI and F-18-fluorodeoxyglucose positron emitting tomography (^18-^FDG-PET). Mini Mental State examination (MMSE) and Hoehn & Yahr (H&Y) stage were used to evaluate cognitive function and disease progression, respectively. Clinical features of the patients are summarized in Table 1. For detailed clinical history, see Additional file 1.

**Table 1:** Summary of the demographic, clinical and neuroimaging information of the study subjects

Patient ref no.	Family 66			Family 97
	66-E	66-K	66-L	97-E
Gender/AAE (y)	Female/35	Male/28	Male/31	Female/30
AAO (y)	26	22	23	25
Initial symptoms	Depression/bradykinesia	Depression/tremor	Bradykinesia	Neuropsychiatric symptoms
Bradykinesia	+++	++	+++	++
Tremors	Yes	Yes	No	No
Postural instability	+++	++	+++	+++
Dystonia	No	No	No	No
Loss of ambulation	3 y after onset	ambulatory	3 y after onset	2 y after onset
Pyramidal signs	++	+	++	+
Cerebellar signs	No	No	No	No
Levodopa response	Yes -minimal	Yes -minimal	Yes -minimal	Yes -moderate
Levodopa-induced dyskinesia	++	–	++	–
Frontotemporal atrophy on MRI	++	+	++	++
Iron accumulation	No	No	No	No
PET scan	Frontotemporal lobe	Frontotemporal lobe	Not done	Frontotemporal lobe
Hoehn & Yahr stage	4.5	4	5	4
Neuropsychiatric symptoms	++	++	+	+++
Emotional Liability	+++	+	+	+++
Cognitive decline	+	++	+	+
Semantic disorder	+	+	+	–
Autonomic symptoms	Yes	Yes	Yes	No
Bladder disturbances	Urgency	Urge incontinence	Urgency	–
Sweating	Yes	Yes	No	–
Flushing	No	Yes	No	–
Sleep disorders	Yes	Yes	Yes	Yes
REM-sleep behavior disorder	Yes	No	No	–
Vivid dreaming	No	Yes	No	Yes
Sleep fragmentation	Yes	Yes	Yes	–
Death	Alive	Alive	Died after 8 y	Alive

*AAE* age at examination; *y* years; *AAO* age at onset; *REM* rapid eye movement; *+* mild; *++* moderate; *+++* severe

### Targeted-NGS

Pathogenic mutations in parkinsonism-related genes, such as *PARKIN* [MIM 602544], *PINK1* [MIM 608309], *DJ*-*1* [MIM 602533], *SNCA* [MIM 163890], *LRRK2* [MIM 609007], *UCHL1* [MIM 191342], and *FBXO7* [MIM 605648], had previously been excluded in all index cases by means of Sanger sequencing before undergoing targeted-NGS [5]. Custom Neurology panel, developed by the Saudi Mendeliome Group as part of the Saudi Human Genome Program [6], was utilized to evaluate peripheral blood DNA from the probands of both families. The panel includes 758 OMIM-listed genes implicated in neurological disorders. Library building, NGS, and bioinformatics analysis was carried out as previously described [6]. Short-listed variants (those that passed the filtering criteria) were validated by Sanger sequencing and subsequently screened in available affected and unaffected family members.

### Genotyping

Parents and the index case from each family were genotyped using the Affymetrix Axiom Arrays according to the manufacturer’s recommendations (Affymetrix, Santa Clara, CA 95051, USA), genotypes were called using Genotyping Console™ (GTC version 4.2), and generated files were further integrated for markers flanking the *PLA2G6* locus; 72 markers were selected in the haplotype analysis. In addition to the Axiom analysis, conventional genotyping was performed using five previously reported microsatellite markers within chromosome 22 flanking *PLA2G6* (*D22S426*, *D22S1045*, *D22S445*, *D22S1156*, *D22S423*) [7]. The PCR amplicons were electrophoretically separated on ABI Prism 3100 Genetic Analyzer and the data was analyzed using GeneMapper 5 (Applied Biosystems, Foster City, CA, USA).

## Results

### Mutation detection and haplotype analysis

**Fig. 1:**
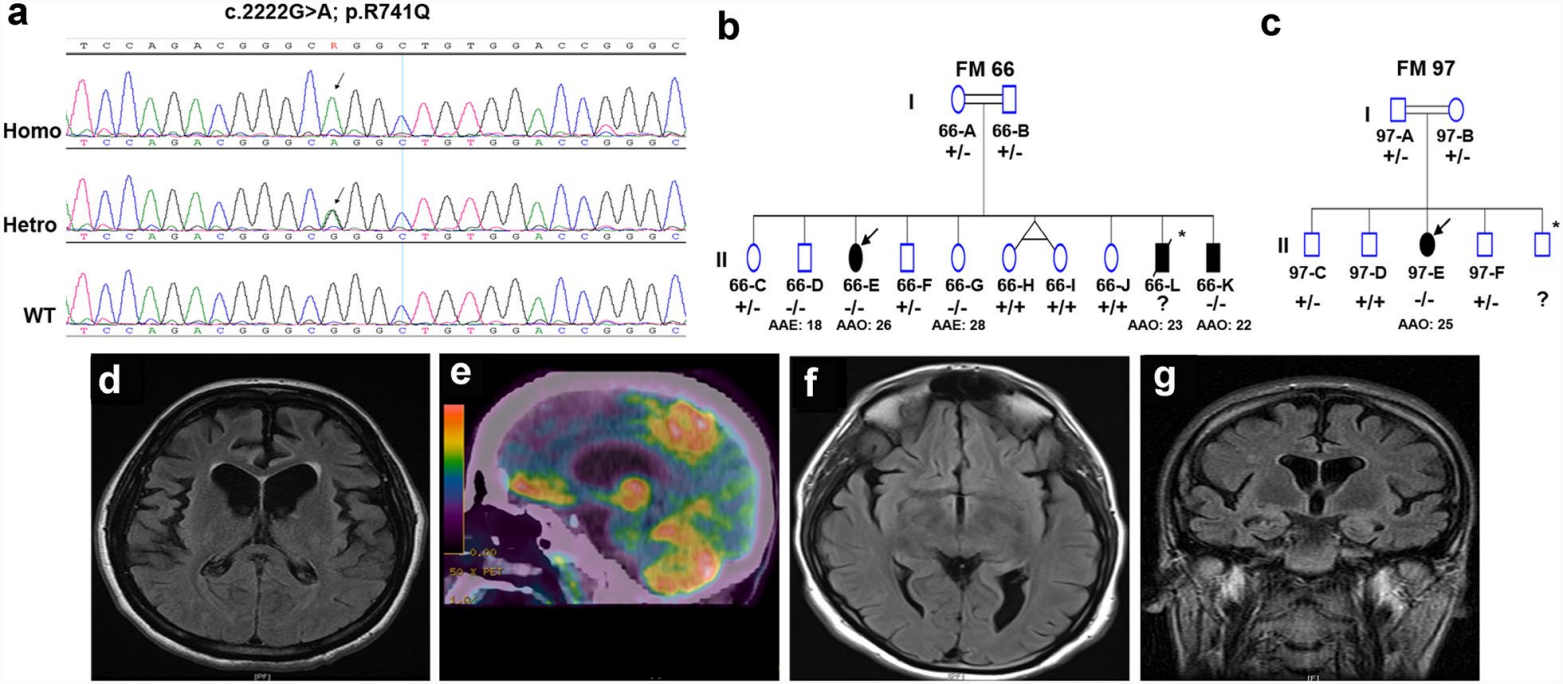
p.R741Q mutation of *PLA2G6* detected in two families with early-onset parkinsonism. **a** Chromatogram of the c.2222G > A mutation. **b**, **c** Pedigrees of the two families showing the genotypes of the mutation. **b** Pedigree of family 66 (FM 66). **c** Pedigree of family 97 (FM 97). **d**–**g** Example radiological imaging of the affected individuals. **d**, **f** Axial brain fluid attenuated inversion recovery (FLAIR) MRI sequence of the frontotemporal region of 66-E (**d**) and 66-K (**f**) showing moderate (**d**) and mild (**f**) atrophy. **e** ^18-^FDG-PET scan of 66-E showing moderate decrease in glucose uptake in the frontoparietal regions. **g** Coronal FLAIR MRI sequence showing moderate frontotemporal atrophy of 97-E. * denotes no DNA sample available. *AAE* age at examination in years; *AAO* age at onset in years

Targeted-NGS analysis revealed a previously described *PLA2G6* homozygous mutation [c.2222G > A (p.R741Q)] [8] (Fig. 1a) shared by both probands (66-E and 97-E) as well as an affected sibling (66-K) (Fig. 1b, c). The mutation was absent in the 1000 Genomes Project database, in addition to our in-house Saudi human genome database (~1000 controls), and was confirmed by bidirectional Sanger sequencing. We next sought to assess the segregation of this mutation, and, to that end, DNA samples from affected and unaffected family members of both families (FM 66 and FM 97) were screened for the p.R741Q mutation. The mutation appears to segregate with the disease in FM 97 as clinically unaffected members were either heterozygotes or wild-type (Fig. 1c). As for FM 66, the presence of this homozygous mutation in two asymptomatic members (66-D and 66-G) is suggestive of incomplete penetrance (Fig. 1b).

**Fig. 2:**
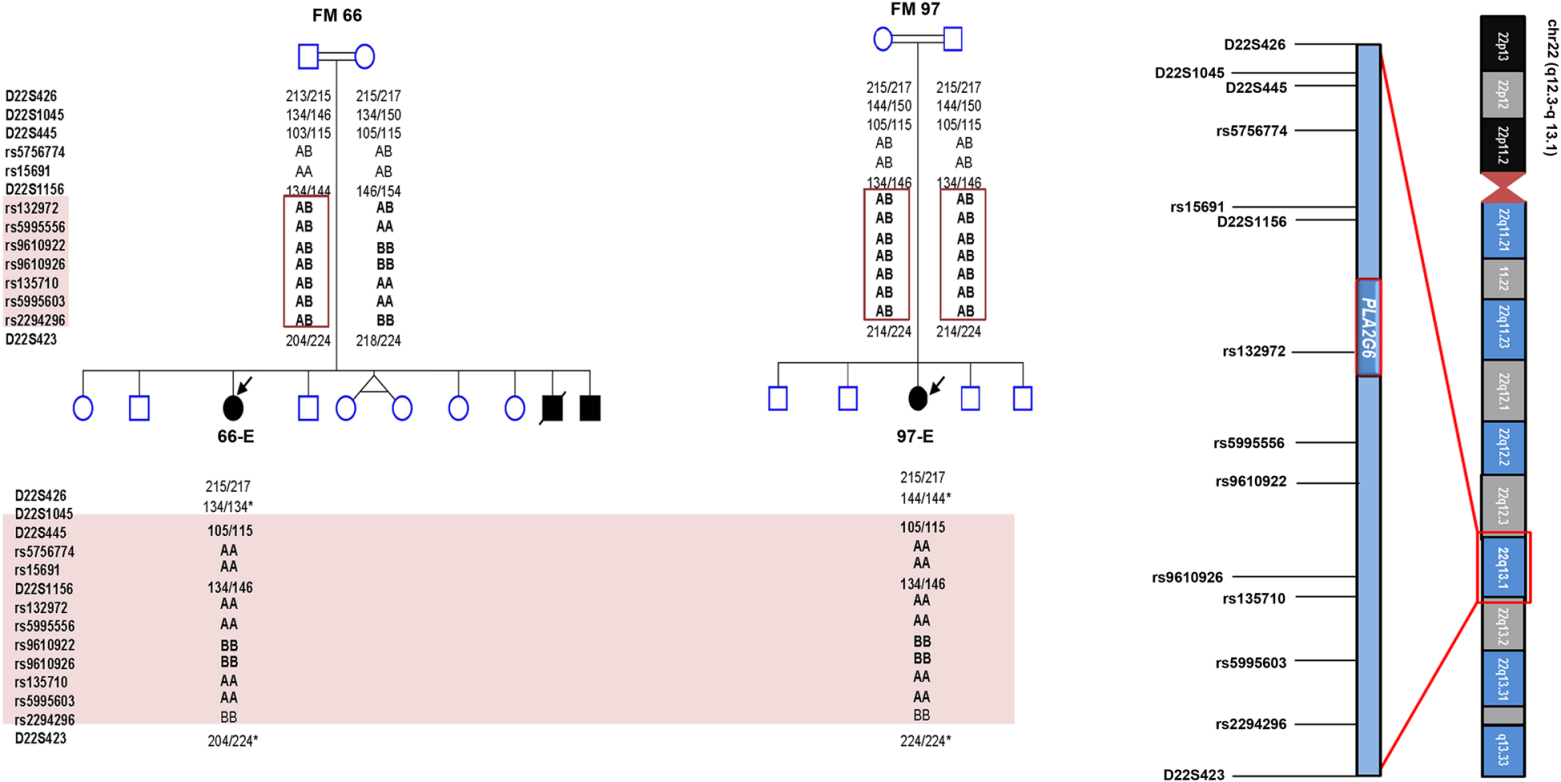
Haplotype analysis of the two families suggests a connection to a common ancestor. The Father of 66-E and the parents of 97-E carry a common haplotype extending 7 markers (*top*). The probands (66-E and 97-E) from both families share the same haplotype for 11 (SNP/STS) markers (shaded in pink) flanking *PLA2G6* on Chr22 including one intragenic (rs132972) (*bottom*). Schematic representation of Chr22 adapted from UCSC genome browser showing the position of some of the markers included in the analysis (*right*). * The different genotypes could be due to recombination events

Since both families originate from the same geographical area and share a specific mutation, we suspected them to have descended from a common ancestor. This notion was examined by genotyping 72 SNP markers along with five previously described microsatellite markers [7] spanning a 3.3 Mb segment on chromosome 22 harboring the *PLA2G6* locus. The analysis revealed a shared haplotype between the father of 66-E and the parents of 97-E spanning a 0.49 Mb segment on chr22q13.1 containing part of *PLA2G6*. The probands, on the other hand, shared the same haplotype across a larger region (1.4 Mb) (Fig. 2). Overall, the genotyping results suggest that the p.R741Q mutation is likely to be inherited from a common ancestor.

### Clinical features of the patients homozygous for p.R741Q mutation

At the age of 26 years, the proband of FM 66 (patient 66-E) presented with bradykinesia, tremors, neuropsychiatric symptoms, and sleep disturbances. A year later, she became almost anarthric, using sign language, and was confined to a wheelchair due to poor balance. She showed marked generalized rigidity affecting axial more than appendicular muscles; however, no dystonia was observed. During her admission, she was started on Levodopa/Carbidopa therapy. This caused a stereotyped, predictable episode of agitation, crying and moaning with semi-rhythmic dyskinetic and dystonic movement-including craniofacial dystonia- with clenching of hands, scratching, and mutilating movements. These movements would start 30–40 min after a Levodopa dose and last for 1.5–2 h. This occurred even with the smallest possible dose of Levodopa, i.e. 50 mg/day. Alternatively, the patient was switched to dopamine agonist therapy in the form of Pramipexole. The dose was gradually increased to 3 mg/day with some improvements in the bradykinesia; her walking improved moderately, but she still required assistance with standing and walking. However, her agitation and emotional lability did not match the symptomatic motor improvement. MRI brain scan showed moderate atrophy in the frontotemporal region (Fig. 1d) with absence of iron deposition in the basal ganglia which was confirmed by T2* sequence. Dopamine transporter single-photon emission computed tomography (DaT-SPECT) scan revealed symmetrical reduced uptake bilaterally in the basal ganglia (image not available) and ^18-^FDG-PET showed moderate decrease in glucose uptake in the frontoparietal regions (Fig. 1e).

At the age of 22 years, the affected sibling of the proband (patient 66-K) started showing signs of motor dysfunction, including bradykinesia, poor balance, and symmetrical tremors, that led him to have three road traffic accidents in one year. Signs of autonomic dysfunction, sleep disorder, and psychiatric problems were reported. Brain MRI (Fig. 1f) and PET imaging revealed similar but milder findings compared to the proband (66-E). Moderate cognitive deficit was shown on neurological assessment. The patient manages limited daily activities on a combination therapy of dopamine agonist, amantadine, MAO-Inhibitors, and atypical antipsychotic. The proband also has another affected sibling (patient 66-L-deceased) that was a former patient at KFSHRC. See Additional file 1 for detailed clinical history.

The proband from the second family (97-E) was leading a normal life as a full time nurse until the age of 25 when she developed neuropsychiatric symptoms. A few months later, motor symptoms, such as rigidity, bradykinesia and poor balance, were evident. The initial diagnosis was drug-induced Parkinsonism, and, subsequently, she was treated with anticholinergic and electroconvulsive therapy. She became relentlessly dependent, needing two people to help her stand up and walk on her tip-toes. The examination revealed severe Parkinsonism (H&Y stage >4.5) with mild cognitive impairment (MMSE score of 18). Brain MRI showed moderate frontotemporal lobar atrophy (Fig. 1g) with no iron deposition in the basal ganglia and ^18-^FDG-PET scan showed bilateral frontal and parietal reduction of glucose metabolism. Like in the previous cases (66-E and 66-L), Levodopa therapy triggered adverse emotional–side effects in this patient. She had frequent episodes of loud crying, tearing, clenching of the mouth at times or bringing her head and neck backward, refusal to eat, self-scratching and stereotyped repetitive movements involving the arms and legs. She became more disturbed after Levodopa therapy and developed craniofacial dystonia and, consequently, was started on a combination of second line medication, including Clozapine, Amantadine, Clonazepam and Pramipexole.

## Discussion

Here we report on the clinical and neuroimaging findings of patients from two different families presenting with early-onset Parkinsonism in whom the reported *PLA2G6* mutation (c.2222G > A; p.R741Q) [8] was detected. The two families, with no reported relationship, originate from the Eastern province of Saudi Arabia; however, haplotype analysis suggests that the mutation may be inherited from a common ancestor (Fig. 2).

The clinical features of our patients overlap with what have been reported in the original cases harboring the p.R741Q mutation [8] with the exception that there was no dystonia reported. Additional features, including sleep and autonomic problems, were also noticed (Table 1). Of note, response to levodopa was limited by the adverse emotional side-effects.

All recruited patients, two of which are siblings (66-E and 66-K), were born to consanguineous parents (Fig. 1b, c). The autosomal recessive transmission of this mutation, as demonstrated by the genotypes and pedigrees, is consistent with the first report of this mutation in a Pakistani/Indian family and with other reports on different *PLA2G6* mutations in PARK14-linked Parkinsonism patients from other populations [3, 8]. The parents of 97-E were heterozygous carriers and the siblings were either heterozygotes or wild-type; they all have passed the expected age of disease-onset at the time of examination without showing any symptoms (Fig. 1c) which suggests that the mutation co-segregates with the disease as previously reported [8]. Unlike FM 97, the mutation does not appear to segregate with the disease in FM 66 as not only the affected individuals were homozygous carriers, but also two asymptomatic individuals; one (66-G) above and the other (66-D) under the age of disease-onset reported in the proband (Fig. 1b). Defining the penetrance of *PLA2G6* mutations in PARK14-linked Parkinsonism patients is complicated by the age of onset, that can vary widely among patients (8–37 years) and within families [3, 8, 9], which could lead to pseudo-incomplete penetrance. Pseudo-incomplete penetrance is a term used when an inaccurate assumption of non-penetrance has been made due to incomplete clinical examination or absence of symptoms at the time of examination [10]. Moreover, asymptomatic homozygous mutation carriers may exhibit preclinical signs; for instance, Shi and colleagues described a slightly reduced uptake in the right posterior putamen of a homozygous carrier of p.D331Y mutation in *PLA2G6* who was clinically unaffected at the time of examination [9]. In this regard, including DAT imaging to the initial clinical assessment and following-up asymptomatic homozygous mutation carriers should warrant accurate penetrance determination.

*PLA2G6* is ubiquitously expressed with widespread presence in all areas of mammalian brain [11]. Its product, iPLA_2_-VI, which catalyzes fatty acids hydrolysis from phospholipids and lysophospholipids, plays a key role in maintaining cell membrane homeostasis. The high lipid content of the brain renders the CNS especially sensitive to lipid metabolism dysregulation. For instance, arachidonic acid, a product of PLA_2_ activation, contributes to the generation of ROS which indirectly induce cellular lipid peroxidation compromising membrane integrity and fluidity and/or disrupting permeability and ion homeostasis [2]. These alterations may underlie axonal dystrophy and iron accumulation typical of INAD and NBIA, respectively [11]. Similarly, the connection between defective phospholipid metabolism and Parkinsonism was speculated on the basis of iPLA_2_-VI role in ROS generation and lipid peroxidation. This was supported pathologically by the widespread Lewy body presence that is most pronounced in the neocortex documented in postmortem analysis of patients with *PLA2G6* mutations and clinically by the presence of Parkinsonism features [4].

Distinct *PLA2G6* mutations give rise to various phenotypes and have different effects on iPLA_2_-VI catalytic activity. For instance, impaired enzyme catalytic activity was reported in iPLA_2_-VI proteins extracted from 293FT cells expressing different mutations associated with either INAD or NBIA. However, results on the effect of dystonia-parkinsonism associated mutations were controversial. Engel et al. [12] reported normal catalytic activity of recombinant iPLA_2_-VI proteins containing missense mutations identified in dystonia-parkinsonism patients, including the p.R741Q substitution reported herein, while another study reported a reduced catalytic activity of the recombinant enzyme containing each of the (novel) mutations identified in Chines Han PD patients [13]. Although p.R741Q and other dystonia-parkinsonism associated mutations were shown not to alter the enzyme catalytic activity, their interference with substrate recognition or other regulatory elements of iPLA_2_-VI remains possible. Moreover, the discrepant findings could be explained by the fact that different mutations, not only within the same gene but also at the same amino acid residue, could elicit different effects on the protein function. This notion is further illustrated by the differential effects of substituting Arg741 residue with either Trp or Gln on iPLA_2_-VI catalytic activity and phenotypic outcome [12].

## Conclusion

In conclusion, we described a homozygous p.R741Q mutation in *PLA2G6* in 3 affected individuals from two families with clinical features resembling dystonia-parkinsonism. Six different homozygous *PLA2G6* mutations were previously identified in 11 patients from 6 unrelated consanguineous Saudi families with variable phenotypes. Out of the 11 individuals, 6 presented with INAD, 4 had a phenotype resembling Karak-syndrome, and one presented with atypical NAD [14]. Our patients presented a phenotype different from what was reported by Salih and colleagues [14], despite their matching ethnicity. This emphasizes the clinical heterogeneity of *PLA2G6* mutations not only across populations but also within a single population.

## Additional file

10.1186/s13104-016-2102-7 Detailed clinical history of the patients.

## Abbreviations

Arg: arginine
CNS: central nervous system
DaT-SPECT: dopamine transporter single-photon emission computed tomography
^18-^FDG-PET: F-18-fluorodeoxyglucose positron emitting tomography
FLAIR: fluid attenuated inversion recovery
Gln: glutamine
H&Y: hoehn & yahr
INAD: infantile neuroaxonal dystrophy
iPLA_2_-VI: calcium-independent group VI phospholipase A2
IRB: Institutional Review Board
KFSHRC: King Faisal Specialist Hospital and Research Center
MAO: monoamine oxidase
MMSE: mini mental state examination
MRI: magnetic resonance imaging
NBIA: neurodegeneration with brain iron accumulation
NGS: next generation sequencing
OMIM: online Mendelian inheritance in man
PD: Parkinson’s disease
RAC: Research Advisory Council
ROS: reactive oxygen species
Trp: tryptophan
