# Supplementary material for: Clinical heterogeneity of PLA2G6-related Parkinsonism: analysis of two Saudi families
Source: BMC Res Notes. 2016 Jun 7;9:295. doi: 10.1186/s13104-016-2102-7 (PMC4897907; doi:10.1186/s13104-016-2102-7)
Supplement: Supplementary file 1 — 10.1186/s13104-016-2102-7 Detailed clinical history of the patients. [file 13104_2016_2102_MOESM1_ESM.docx]

**Supplementary Information**

**Detailed clinical history of the patients**

**FM 66**

*66-E*

A 35 year old native Arab woman originating from the Eastern province of Saudi Arabia, this patient is the product of a consanguineous marriage (Fig. 1B). Up until the age of 26, she was normal; she got married and had a child at age 22. At age 26, over a few months, she developed gradual onset bilateral symmetrical tremors, bradykinesia, sleep fragmentation, visual and auditory hallucination, personality changes with uncontrolled anxiety, imbalance,clumsiness, and falling backward along with dysarthria and difficulty swallowing. A year later, she became almost anarthric, using sign language, and was confined to a wheelchair due to poor balance. She had facial hypomimia and hypometric horizontal saccade with no other ocular motility problems. Fundus examination was normal with no Kayser-Fleischer rings. She was severely dysarthric with decreased oral motor skills. Retraction of the back of the tongue was observed with severe delay in swallowing initiation, decreased laryngeal elevation, and decreased posterior pharyngeal wall contraction leading to repeated silent aspiration with thin nectar and puree food. She showed marked generalized rigidity affecting axial more than appendicular muscles; however, no dystonia was observed. Intermittent asymmetrical 5-6 Hrz pill rolling rest more than postural tremor. Power was normal with marked hyper reflexia and ankle clonus and extensor planter responses. Bilateral limb bradykinesia and motor impersistence with attempt to make a stand; she was able to walk a few steps on her toes when she was assisted and supported to stand. If unsupported, she would fall back spontaneously.

During her admission, she was started on Levodopa/Carbidopa therapy. This caused a stereotyped, predictable episode of agitation, crying and moaning with semi-rhythmic dyskinetic and dystonic movement –including craniofacial dystonia- with clenching of hands, scratching, and mutilating movements. These movements would start 30 to 40 minutes after a Levodopa dose and last for 1.5 to 2 hours. This occured even with the smallest possible dose of Levodopa, i.e. 50mg/day. Alternatively, the patient was switched to dopamine agonist therapy in the form of Pramipexole. The dose was gradually increased to 3 mg/day with some improvements in the bradykinesia; her walking improved moderately, but she still required assistance in standing and walking. However, her agitation and emotional lability did not match the symptomatic motor improvement. A percutaneous gastrostomy tube was inserted because of difficulty in swallowing. All blood tests were normal; cerebrospinal analysis showed a decrease in homovanilic acid but was otherwise normal. MRI brain scan showed moderate atrophy in the frontotemporal region (Fig. 1D) with absence of iron deposition in the basal ganglia which was confirmed by T2* sequence. DaT-SPECT, done outside our institution, revealed symmetrical reduced uptake bilaterally in the basal ganglia (images not available) and ^18-^FDG-PET showed moderate decrease in glucose uptake in the frontoparietal regions (Fig. 1E). The scan also showed global reduced metabolic activity involving most of the cerebrum more severe on the right side with mild sparing of the high parietal lobes and relatively spared basal ganglia and thalami (Fig. 1E). Visual evoked potentials and neurophysiological examinations were normal.

*66-K*

A 28 year old single male was normal until the age of 22, when he developed a fairly rapid onset of difficulty with balance and walking, symmetrical tremors, and slowness in his movements that led him to have 3 road traffic accidents in one year. His sleep became disturbed and fragmented. He became easily irritable with episodes of sweating, urge incontinence, and sudden emotional outburst. He developed a Serotonin syndrome when he was on SSRI medication. Over the ensuing few months, his tremor became apparent with impulsive behavior; he was difficult to control andstarted to have choking episodes. Brain MRI (Fig. 1F) and PET imaging revealed similar but milder findings compared to the proband (66-E). A neuropsychological assessment showed moderate widespread cognitive dysfunction; in particular, he had a severe frontal executive dysfunction. He scored 50% in verbal and visual memory. His verbal IQ was 72 with difficulties in word retrieval. There was moderate rigidity, bradykinesia, asymmetrical tremor, and impaired postural reflexes. He was still ambulatory with very limited daily activity on Dopamine agonist, amantadine, MAO-Inhibitors, and atypical antipsychotic.

*66-L*

Patient was seen previously; his medical report indicated that his disease started at age 23 with behavioral changes, mild anxiety, and depression. Bradykinesia started early and was treated with anti-psychotic and anti-depressant. A few months later, his postural imbalance progressed. He did not respond to anti-psychotic and anticholinergic drugs. He became bed-bound 3 years into his illness. Levodopa therapy caused episodes of screaming, crying and abnormal postures. Brain MRI showed moderate frontotemporal lobar atrophy. He had marked difficulty in swallowing. The family refused gastrostomy tube feeding, and he passed away after an episode of aspiration pneumonia.

**FM 97**

*97-E*

A female seen at age of 30, the eldest among 4 other siblings. Parents are first degree cousins; this family also originated from the same region in Saudi Arabia, but no relation to FM 66 was reported. Patient was working as a full time nurse until the age of 25 when she started getting depressed, crying, and becoming socially withdrawn; she was paranoid believing that her food and water were poisoned. She became pre-occupied with visual hallucinations with vivid dreams and frequent moaning; she was started on various antipsychotics for diagnosis of schizoaffective disorder. A few months later, she started to get stiff and slow having trouble with her balance when walking – walking backwards until she hit something She showed bradykinetic drooling with dysphagia. She was initially diagnosed with drug-induced Parkinsonism and was treated with anticholinergic and electroconvulsive therapy. She became relentlessly dependent needing two people to help her stand up and walk on her tip-toes. On examination, she can follow simple commands and mumble a few words. In the MMSE, she scored only 18 points. She kept her head held backward with axial rigidity. She had hypometric saccadic ocular movement. The lingual and labial movements with resting tongue tremors with weak cough gag reflex. She had moderate limb paratonia, axial more than limb rigidity with retrocollis. H&Y stages were over 4.5 - even in the best on period. There was mild distal weakness with hyperreflexia, with no clonus and flexor planter response. The brain MRI showed moderate frontotemporal lobar atrophy (Fig. 1G) with no iron deposition in the basal ganglia and ^18-^FDG-PET scan showed bilateral frontal and parietal reduction of glucose metabolism. During her stay in the hospital, she had frequent episodes of loud crying, tearing, clenching of the mouth at times or bringing her head and neck backward, refusal to eat, self-scratching, and stereotyped repetitive movements involving the arms and legs. She became more disturbed after Levodopa therapy and developed craniofacial dystonia and, consequently, was started on a combination of second line medication, including Clozapine, Amantadine, Clonazepam, and Pramipexole.
